# First do no harm: practitioners’ ability to ‘diagnose’ system weaknesses and improve safety is a critical initial step in improving care quality

**DOI:** 10.1136/archdischild-2020-320630

**Published:** 2020-12-23

**Authors:** Mike English, Muthoni Ogola, Jalemba Aluvaala, Edith Gicheha, Grace Irimu, Jacob McKnight, Charles A Vincent

**Affiliations:** 1 Oxford Centre for Global Health Research, Nuffield Department of Medicine, University of Oxford, Oxford, Oxfordshire, UK; 2 Health Services Unit, KEMRI-Wellcome Trust Research Programme, Nairobi, Kenya; 3 Department of Paediatrics and Child Health, University of Nairobi, Nairobi, Kenya; 4 Kenya Paediatric Research Consortium, Nairobi, Kenya; 5 Experimental Psychology, University of Oxford, Oxford, UK

**Keywords:** health services research, neonatology, nursing care, data collection

## Abstract

Healthcare systems across the world and especially those in low-resource settings (LRS) are under pressure and one of the first priorities must be to prevent any harm done while trying to deliver care. Health care workers, especially department leaders, need the diagnostic abilities to identify local safety concerns and design actions that benefit their patients. We draw on concepts from the safety sciences that are less well-known than mainstream quality improvement techniques in LRS. We use these to illustrate how to analyse the complex interactions between resources and tools, the organisation of tasks and the norms that may govern behaviours, together with the strengths and vulnerabilities of systems. All interact to influence care and outcomes. To employ these techniques leaders will need to focus on the best attainable standards of care, build trust and shift away from the blame culture that undermines improvement. Health worker education should include development of the technical and relational skills needed to perform these system diagnostic roles. Some safety challenges need leadership from professional associations to provide important resources, peer support and mentorship to sustain safety work.

**What is already known on this topic?**

- Harm resulting from unsafe care is common and results in significant adverse health and economic consequences in high-income countries.
- Efforts to prevent or reduce harms often focus on identifying errors so that their specific causes can be addressed.
- More recently, attention has been turned to considering how harms arise as a product of complex interactions in systems.

**What this study adds?**

- Patient safety is much less well studied in low-resource settings than in higher income settings.
- We suggest how concepts being employed to advance patient safety thinking in higher income settings could be usefully applied by practitioners in low-resource settings.
- The ability to diagnose system weaknesses should become a core skill for those leading teams, wards, departments or facilities in low-resource settings.

## Introduction

Healthcare systems across the world are under pressure and often unable to meet the standards of care articulated in international and national policies. The gaps are acute in low-resource settings (LRS) and the need to strengthen health systems is now well recognised.^1^ Promoting safer care to avoid harm as part of this agenda is an important goal of the WHO.^2^ However, we now recognise that healthcare delivery is a system characterised by multiple interacting elements, or complexity.^3^

In complex systems, the effects of any intervention may defy simple linear logic and even be counterintuitive.^3 4^ This discussion of systems may seem remote from the day to day tasks of health workers. However, even a hospital ward is a system with interdependent elements forming a complex whole. We argue that health workers have key ‘insider’ insight how these systems work but they need to develop skills and be mentored so that they can ‘see’ or critically explore these systems to improve patient safety.^5^ Such system diagnostic skills are as important as good clinical skills, especially for those with team, department or facility management responsibilities. To illustrate this, we focus on how leaders in LRS hospitals can identify local safety concerns to benefit their patients. To do this, we use the example of a Newborn Unit (NBU) to explore some of the ‘diagnostic’ approaches that can be applied. We believe there is also a role for research to promote shared learning and larger scale change based on evidence. We deal with strategies that may be used to address safety problems in an accompanying paper.^6^

## Seeing and appraising local care as a system

### A systems framework

NBUs in LRS hospitals are high-pressure ‘critical care’ settings. As a new clinical or nurse leader enters such an NBU (box 1) what will their first impressions be? Will they simply see business as usual, or will they already begin to appraise the challenges of providing safe care and how they might prioritise and implement solutions? Are they aware of what they are expected to manage or their options for exerting influence? Might they reach out to peers and mentors for support and to extend their circle of influence to develop broader, collective solutions?

Box 1 **Brief description of a low-resource settings Newborn Unit that provides care to low-income families for those unfamiliar with these settings**

Mothers, some who gave birth less than 24 hours ago, may be bewildered, anxious and exhausted. They spend time with their babies in settings that are often crowded and seemingly chaotic. Staff may compete to access and use poorly organised patient files, junior doctors often conduct mini-rounds in different ward areas while one nurse per room often works independently around them.

Often an ‘acute room’ is home to the most severely ill. Plastic chairs where mothers sit occupy the spaces between incubators together with oxygen cylinders and the occasional box-like oxygen concentrator. Drip stands swing precariously, intravenous lines run into incubators tangled with oxygen tubing. Incubators may accommodate two babies side by side. Emergency care for the newest admissions takes place in the same space under overhead heaters. Materials such as boxes of syringes, needles, bottles of iv fluids and assorted other consumables are stacked on tables. There are no beeping monitors and incubator alarms are typically turned off as many staff have no training in how to adjust them.

Less sick newborns may be cared for in rooms lined with open cots with electric heaters and permanently closed windows to keep the whole room warm. Many still need oxygen, intravenous fluids, phototherapy and intravenous antibiotics. Mothers struggle to express breast milk to pour into syringe chambers attached to nasogastric tubes. The very few qualified nursing staff devote their time to activities such as giving intravenous medicines delegating to students who spend only a few weeks on the ward tasks such as conducting observations and communicating with mothers. There is no time for effective supervision. Doctors own medical records, nurses keep their own, there are often a multitude of poorly filled patient charts that act as forms of communication between doctors and nurses.

Examining the delivery of clinical care is becoming more familiar to many in LRS as quality improvement (QI) approaches drawing on Donabedian’s framework become more widely disseminated.^7^ This focuses attention on how inputs are transformed by processes to outcomes. In the classical diagnostic phase of QI, root causes of problems are identified. Improvement efforts then focus on addressing these, measuring change and adapting or reinforcing actions in cycles until desired levels of performance are reached. While these strategies can be successful in LRS, it can be hard to build and sustain QI teams, gather data and measure performance.^8 9^ The linear, logical approach may also result in improvement efforts focusing on a narrow set of root causes when problems have more complex origins or may constrain improvement targets to only those that can be measured.^10^

Here, we draw on concepts from the safety sciences that are less well-known in LRS to propose complementary strategies for analysing the strengths and vulnerabilities of systems. Specifically, we draw on the Systems Engineering Initiative for Patient Safety (SEIPS) models that have been developed over more than a decade to explore work system domains and how they interact to produce outcomes.^11–13^ Staff, patients and families are all key ‘working parts’ of the system and so all their roles must be considered. Their experiences are also critical outcomes, including their well-being and satisfaction.^14^ The SEIPS framework, like all models, aims at being a guide when thinking about and attempting to simplify complex phenomena and we use it to exemplify and structure an initial approach to system diagnosis by local leaders. It introduces concepts from broader systems thinking concerning the hardware (technology, tools, resources and physical environment) and the visible and invisible software of health systems (for those interested in these wider systems ideas see the study by de Savigny and Adam^15^). Things that are visible include the tasks and established organisational routines that you can see if you observe an NBU or another ward carefully (eg, the process of conducting a drug round). Things that are invisible include the unwritten rules that underlie the habits of staff, which may be quite particular to a ward or hospital. For example, all mothers may leave the ward at the start of the doctor’s round as all have learnt this is expected. When you ask people why this happens, the response is often ‘it is just the way we do things here’. These invisible rules can be very powerful influences and changing them can be difficult.^16 17^ Another example might be the long tradition of using physically and organisationally separate medical and nursing records rather than some form of shared record that might promote information sharing. Separate records may be so ingrained in the nursing and medical professionals’ daily life that it may take considerable effort to engage everyone in examining ‘is this the best way to do things’? All of these local influences are of course embedded in the formal rules, cultures and habits of the wider health system. Importantly, the SEIPS and other systems frameworks remind us that it is the interactions between all elements that result in the care we provide and its outcomes.

### Examining a local system

Let us now think again of a new clinical or nurse leader. After a few weeks of engaging in hands-on care, she may be more familiar with the NBU’s patterns of work. She may return after a weekend off-duty to find a distressed mother, a sense of unease on the ward and a baby who had been doing well who has deteriorated markedly. Over the next few days, the baby continues to deteriorate and dies. Such events may be subject to local clinical audit. This process can be superficial, may result in apportioning blame, creating fear and undermining relationships, and may only identify a limited set of root causes if not well conducted (eg, inadequate staffing).^18^ Often the team providing care also feel the root causes identified are beyond their control.

Good clinician training should, however, accustom us to use a variety of strategies to ensure our diagnosis is complete. What is good practice when dealing with patients’ needs to be applied to our care systems. There are many possible approaches but here we briefly outline three.

#### Reflecting on a case and using a systems view

Cases have always been used to educate, although predominantly on the nature of disease, the process of clinical decision making and the weighing of treatment options. Incident or case analysis can also be used for the purposes of improving the safety of healthcare and goes beyond a typical case audit.^19^ It can encompass all the typical educational perspectives but critically also includes reflection on the local and broader system and, particularly when errors are identified, can extend to explore the personal impact of incidents and mishaps on both staff and families.^20^ Such reflection should not aim to simplify and reduce challenges to a small set of root causes but recognise that a chain of events and a wide variety of factors contribute to any outcome. In such a diagnostic approach, information may be gleaned from a variety of sources. Case records and any other relevant documentation are reviewed. Careful and sometimes confidential discussions with key members of staff and even family members might be undertaken to establish the chronology of events and the full range of contributory factors. Such enquiry might radiate out to parts of the system not originally linked to the events being examined. The key questions are: ‘What happened?’ (the outcome and chronology); ‘How did it happen?’ (identifying problems in the process of care) and ‘Why did it happen?’ (the contributory factors). To get beyond only superficial answers given by people who may fear punishment or shame, the person or team conducting this form of enquiry must be fully trusted.

**Figure 1:**
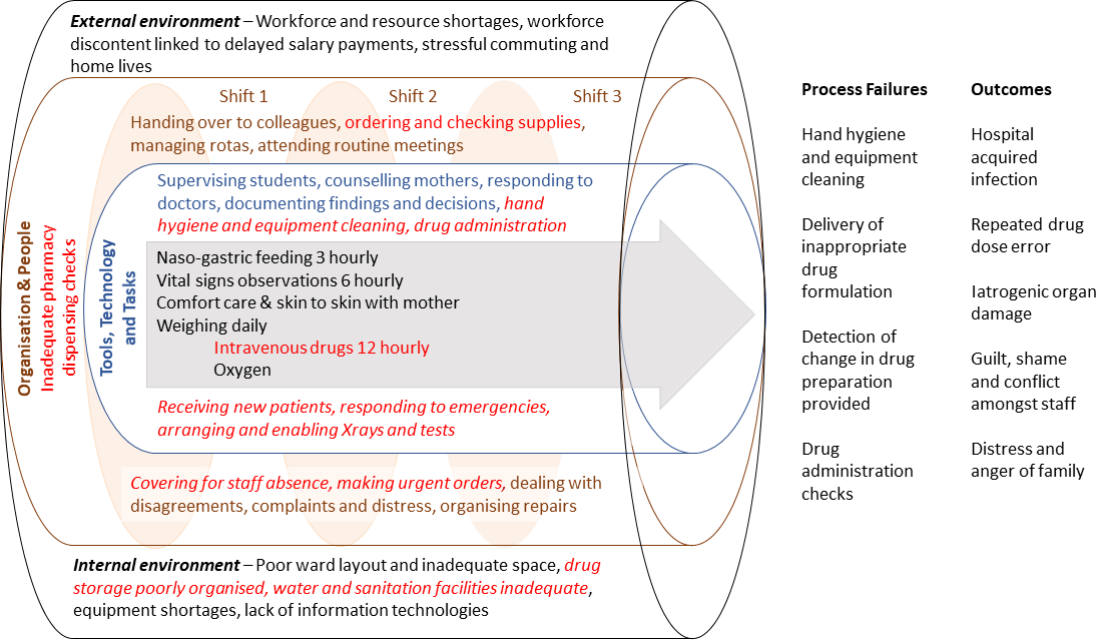
Illustration of how a skilled practitioner might combine their insider knowledge with a more analytical outsider perspective to examine how the external and internal environment, the organisation of work and the different groups of people who conduct tasks, using the tools and technologies available to them, all result in the actual processes and outcomes of care. In this diagram, the inner arrow represents the main care activities, these are often defined on a medical round, as here when baby Bahati was started on a new course of antibiotics and oxygen (shift 1). The Newborn Unit (NBU) team uses tools and technologies (eg, patient monitoring charts, pulse oximeters, weighing scales) and is involved in team-based activities (all encompassed in the blue cylinder). Wider organisational activities also continue (encompassed by the brown cylinder) such as the three shift changeovers. Some of these activities (eg, handing over or ordering supplies) require engagement with other hospital departments or teams. Other activities are less predictable but may strongly influence practice (eg, unexpected staff absence or dealing with a distressed parent). All of this activity takes place within a wider environment (represented by the black cylinder), which can influence NBU care (eg, inadequate supply of medicines or failure to recruit new staff). We highlight using red text how multiple contributing factors that affect baby Bahati’s care (panel 2) combine to produce important process failures and subsequently harms.

We illustrate this approach with an imagined clinical case in box 2 and figure 1. We employ the domains in the SEIPS framework to illustrate how using a more system-oriented model might guide local practitioners to identify multiple problems that interact to produce harm. Building networks to support such work, ideally with mentorship from with those with greater expertise, may then advance our understanding of safety challenges at scale.^21^

Box 2 **Exploring a clinical incident on a low-resource settings Newborn Unit**

The incident: Baby Bahati was born premature weighing 1320g and was in the ‘Acute Room’ for 4 days receiving oxygen for respiratory distress and intravenous fluids. She slowly improved, was receiving expressed breast milk, and was moved to the ‘step down’ room on day 5. By day 9 she was tolerating full breast milk feeds. Her mother and the ward team were visibly happy with her progress. On day 15, after a weekend, Baby Bahati looked much worse. On Friday evening she had developed a fever so antibiotics were started, but she proceeded to develop respiratory distress and vomited several times. Now her abdomen was also distended and she looked a grey colour despite being back on oxygen. Over the next 3 days she continued to deteriorate, stopped passing urine, and eventually died.

Examining the case: Baby Bahati seemed to have succumbed to a hospital acquired infection (HAI), although the hospital could offer no diagnostic tests to confirm this. Attention initially focused on risks for acquiring HAI. The Thursday and Friday before Bahati became unwell had been very busy with new admissions and nursing care in the ‘step-down’ room had largely been delegated to students. In fact, Bahati’s mother had complained on a ward round that these students had not cleaned the scales between weighing each baby and rarely seemed to wash their hands even before inserting a new naso-gastric tube for Bahati. The ‘diagnosis’ of HAI seemed clear, however, two other babies in the acute room had also stopped passing urine, all were receiving gentamicin. Reviewing the treatment sheets, the dose prescribed for Bahati was too high but doses for the other babies were correct. The vials of gentamicin in the drug cupboard were checked. Most vials were those usually supplied with 20mg gentamicin in 2mls, but three remaining from the weekend were an adult preparation and had 80mg in 2mls. There had been an urgent request to the pharmacy on the busy Friday evening for additional gentamicin to tide the ward over the weekend.

System challenges: We can deal only briefly with the diagnostic phase here to illustrate how thinking may be structured using some of the SEIPS domains. (i) Internal environment—poor hand-washing facilities, absence of hand-rub and other materials undermine cleaning of equipment and good hand hygiene, (ii) Organisation—students are relied on to step in and cover workforce gaps at busy times, there are no systems enabling support from additional qualified staff during such periods, and there are no special pharmacy procedures for issuing drugs to the neonatal unit or of checking them on arrival, the system relies on often a single nurse giving medication to make all necessary checks, (iii) Tools and technology—the 20mg and 80mg vials look similar, there are no warnings on the drug cupboard to check the vials and nurses have a single dilution chart that makes reference only to use of 20mg vials, (iv) Tasks—clear procedures for cleaning the weighing scales are not written down, treatment charts are not routinely reviewed over the weekend by a clinician and pharmacists do not conduct ward visits, (v) Patients/Parents—often fear raising concerns with professional staff as they do not want to appear ‘difficult’ and risk unfavourable treatment, (vi) Staff—fearing confrontation, rarely feel confident to suggest other professionals have made mistakes even though all are aware errors are common as people rush tasks to meet the demands of their work.

#### Process analysis: understanding the patient journey

Analyses of specific incidents or cases, especially when systematic and thorough, can illuminate systemic weaknesses and help us understand how things go wrong frequently because of a chain of contributing events. Having understood these principles, we can now approach the examination of system weaknesses from a different perspective. Instead of taking a case and analysing it, we can begin with a process of care and systematically examine it for possible failure points. For example, one might examine the process of handling a preterm birth in a hospital from the point at which the midwives recognise it then through the birth, initial resuscitation/care, transfer to the NBU and stabilisation.

There are many formal methods to guide these kinds of analyses. Here, we consider process mapping that allows us to ‘see’ and understand a task or part of the patient’s journey and experience by identifying the sequence of steps expressed as a pathway.^22–24^ All staff and patients can be involved in the production of a process map and can help identify steps where delays occur, where care might be missed or communication or other problems arise. The end-product is highly visual, easy to understand and an important part of system diagnosis. Any of the following methods can be used to build the process map:

- Multidisciplinary meetings: single or short series of meetings of representative staff in a non-clinical environment
- Walking the journey: following the normal route of the task or patient journey supplemented by patient and staff interviews in the clinical environment
- Direct observation of a task, procedure or patient journey: this can include following a patient’s journey in real time with direct observation and informal interviews
- Patient’s self-reported experience: patients record their experience of the journey in real time.

**Figure 2:**
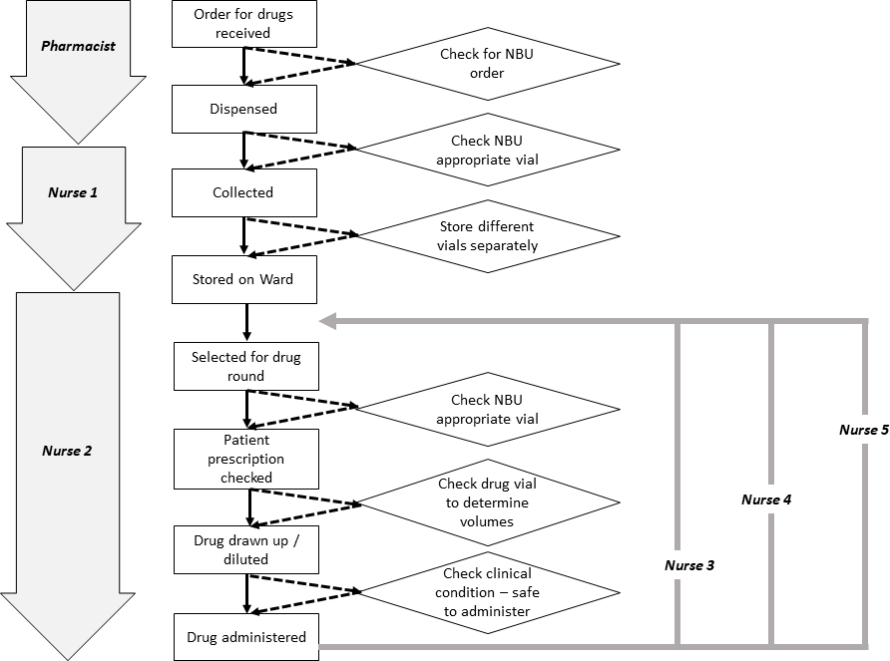
A simple process map representing how drugs are ordered, dispensed and administered in our imaginary Newborn Unit (NBU). Key initial actors (left of figure) include the pharmacist, nurse 1 who collects the drugs and nurse 2 who later gives the first dose of drug. In our example, the process involved a sequence of activities (rectangular boxes connected by unbroken arrows) that took place without any active checking or decision-making steps. We have added potential decision-making or checking steps that would create a new pathway (illustrated by the diamonds and the broken arrows) that six points at which a drug error (panel 1) might have been averted or detected. In our example, as it was not a routine practice to check drug vials for the preparation they contained the drug error was repeated over several days (by nurses 3, 4 and 5).

Such activities need not take a great deal of time and might be conducted as part of continuing medical education schedules, in place of some regular audit meetings or might make use of students to gather data (eg, to track patients). Once a basic map of the process or journey has been produced, then it can be enriched in shorter discussions to identify specific system vulnerabilities or where safety could be improved at key stages of the process. We provide a simple example in figure 2 showing multiple locally feasible opportunities for reducing harm in the case explored above.

#### Mapping strengths and vulnerabilities

As early experience is gained and the team gets more familiar with reflective thinking on systems, they may move beyond a case or specific process or pathway. Ward leaders, working with a few team members, might then use the same SEIPS domains to structure reflections that help them delve deeper into the specific context and characteristics of their local system to identify more general local strengths and weaknesses (table 1). Such thinking may be especially important to surface some of the invisible issues (italic text, table 1). These may be routines or beliefs that are too ‘taken for granted’ to be immediately recognised as problematic. One example we have come across is the practice of making sure some entry is made on patients’ vital signs’ charts at all scheduled times so that a complete chart can be handed over to the next shift. Unfortunately to achieve this, normal measurements are often fabricated giving a false sense of patient well-being. Such practices can often continue unremarked on for years although the entire team is aware of it and individuals therefore have no trust in any observations they do not do themselves. Surfacing such issues, and the reasons for why they arise require staff to trust their leaders and feel psychologically safe.^25^ This might require initial help from a facilitator or mentor who might draw on helpful research in such areas^21^ so that issues can be discussed honestly and context appropriate decisions made. For example, to accept that some missed observations are inevitable but that efforts should focus on monitoring high-risk patients.

**Table 1:** Mapping the strengths and challenges of an LRS NBU and taking a systems perspective based on personal observations and prior research (‘invisible’ issues in italic text)

Domain	Examples of strengths	Examples of challenges
Patients/mothers	Families are highly motivated, are often keen to engage in care once their confidence is built (eg, feeding support and monitoring) and mutually supportive groups including ‘expert’ mothers whose babies have been on the ward the longest. These groups may form especially in hospitals where mothers are resident. Enabling mothers with good information on their baby can help promote continuity of care as they become the bridge between different teams and helps foster the global movement towards family-centred care	May initially be overwhelmed by baby’s illness and have to mobilise resources to supplement those of the hospital (eg, purchasing drugs, feeds and diapers) Levels of literacy and numeracy may limit sharing of care if this is not well designed *Power differentials linked to socioeconomic, educational and cultural factors may undermine relationships between families and staff*
Staff	Many retain a strong vocational commitment to ‘service’ and some experienced nurses have clearly dedicated themselves to NBU work. Committed staff can be champions of safe, high-quality care. Despite resource problems some wards have teams that provide each other with critical social and emotional support—this can be a foundation for further team-based improvements *Staff have considerable knowledge about how the local system works and where key challenges lie, they may have important links to the local community and may be able to mobilise wider support for improvement efforts*	Low staff numbers with high workloads can result in burnout that reduces engagement in improvement efforts Some leaders have not received training in or may lack an appetite for leadership roles Frequent staff turnover Ancillary staff may have poorly defined roles and responsibilities, may be poorly trained (eg, in infection prevention) and little may be invested in their supervision *Fear of blame or confrontation may prevent discussion of mistakes or safety issues* *Interprofessional rivalries may undermine communication, relationships and teamwork*
Tasks, technology and tools	Increase in availability of basic equipment (eg, pulse oximetry, Continuous Positive Airway Pressure, CPAP) Increased access to smartphones or computers and so to knowledge and links with other professionals Considerable innovation already occurring to make efficient use of resources (eg, through task sharing or safe reuse of consumables) *Team has often developed routines that prioritise the most critical tasks to focus on when under extreme time pressure*	Wards may be supplied with the wrong tools (eg, adult drug formulations) or receive new staff with minimal training or experience Staff struggle with poorly designed ‘everyday’ tools, for example, treatment and nursing observation charts inherited unchanged from adult wards *Established routines may suit staff but not optimise patient outcomes and be hard to change* *Adding new technologies gives the impression of more advanced care (giving professionals greater status) but may increase workloads for some staff and so may not improve outcomes*
Teams	Teamwork and respectful communication are highly valued by staff and families The importance of practical experience and learnt skills is recognised in a team not just qualifications or professional cadre *Staff can go out of their way to offer practical or emotional support to other staff and caregivers*	There is little obvious reward or recognition of individuals’ efforts to sustain quality services Practical skills are often developed by individual trial and error, limited attention is paid to coaching and mentorship *Traditional professional hierarchies and poor leadership practices undermine team performance*
Environment and working conditions	Basic infrastructure may be available Many ‘work-arounds’ have been developed that overcome long-standing challenges Relatively small investments/changes can produce substantial benefits *Mothers of babies recognise how challenging things are for staff and are often very positive and grateful when they are well cared for*	The physical space is often poorly suited to needs of NBU (eg, power outlets, oxygen systems) Inadequate attention is paid to basic infection control and staff and family needs (eg, adequate toilets) Overcrowding undermines effective care *Little support is provided to staff and families who may experience emotional distress*
Organisation	There is emerging recognition that safety is reputationally critical and a key part of quality improvement Some senior staff have gained knowledge and skills in how to work within existing local and political systems to effect change *Effective leadership can inspire shared goals and a sense of ‘mission’ despite challenging environments*	There is often a feeling that senior management are only concerned about quality for appearances sake so quality and safety activities are conducted ‘just to tick boxes’ Local leaders have limited control of spending or resources limiting their ability to make changes Hospitals may rely for equipment on donations making it hard to execute a plan for improvement *Hospital leadership may create a fear of being blamed for poor outcomes so there is an unwillingness to acknowledge errors*

LRS, low-resource settings; NBU, Newborn Unit.

### Summary

The exploration of the ward as a system outlined above is, we argue, an important diagnostic skill that needs to be learnt. We and others have previously shown that those taking up clinical management roles may be ill-prepared for such team leadership roles.^26–28^ Building such skills, an issue we return to in an accompanying paper,^6^ might enable local leaders to address key safety issues in their own unit, addressing others might benefit from joining with other practitioners or mentors. Acting locally will require sensitivity and tact. Leaders will need to build trust so team members know they will be fairly treated and not made vulnerable if we are to shift away from the blame culture that is toxic to improvement efforts but common in traditionally hierarchical work settings.^18 25 29^ Emphasising shared experiences and values may be especially important in establishing the conditions to support safer care in high stress LRS hospitals as part of building ‘everyday resilience’.^30^ We believe health worker education must embrace development of the technical and relational skills needed to perform these system diagnostic roles.

The potentially overwhelming list of challenges to be tackled can be daunting (panels 1 and 2, figures 1 and 2, and table 1). It is important to focus initially on practical, safety critical issues that advance the team’s ability to provide the best attainable level of care in a specific context rather than repeatedly fail to meet impossible aspirational standards. As a first step, local teams might create the time to meet and discuss honestly what they believe is the best attainable level of care they can achieve in the short term and identify their priorities for improvements in the medium term. For example, much medical frontline care is provided by rapidly rotating junior staff with very limited specific neonatal training.^31^ Rotation planning, structured periods of orientation, empowering skilled nurses to coach or correct junior physicians and having clear protocols including when to escalate concerns might all support safer care, but these are given little attention in many LRS.

Some safety work may be best tackled by practitioners who work in similar settings coming together as communities, and this need not be costly. For example, public hospital staff might come together to tackle one problem we have seen of a multitude of poorly designed observation, treatment and drug preparation charts. Their poor design promotes errors, means information they contain is often ignored and therefore they often waste considerable staff time. In some cases, tackling safety concerns may need to involve a national professional association working with government, for example, to improve neonatal and paediatric drug procurement or labelling (as in our example) or to create clear guidance on approved equipment.

## Conclusion

First, our efforts to provide care should do no harm. This means practitioners should have the ability to diagnose how multiple system factors can increase the risk of harm. The approaches highlighted can be complementary to traditional QI. However, they emphasise critical analysis and reflection and focus on prevention so that it can be harder to measure impact (eg, by using a run chart). Senior practitioners may need training and mentorship to work with their teams to address the organisational risks that may harm patients, families and staff. In LRS we should recognise, celebrate and learn from those who achieve the best attainable standards of safe and effective care as teams and with families.
